# In-process large language model requesting through knowledge graphs for interfacing with manufacturing digital twins

**DOI:** 10.1088/3049-4761/aea41f

**Published:** 2026-09-21

**Authors:** Reiki Tanaya Karuka, Vinh Nguyen

**Affiliations:** Department of Mechanical and Aerospace Engineering, Michigan Technological University, Houghton, MI, United States of America

**Keywords:** digital twin, large language models, knowledge graph, 3D printers, in-process monitoring

## Abstract

Digital twins (DTs) have potential to improve manufacturing processes by providing in-process monitoring and reducing trial-and-error while improving process prediction and performance. However, identifying anomalies or defects in a DT environment remains a very time intensive process that requires multidisciplinary expertise and significant manual effort in analyzing the system. Large language models (LLMs) offer potential capabilities to interpret system-level information and support user-driven querying, but their effectiveness is limited by hallucinations when reasoning over large and dynamic manufacturing datasets inherent to process monitoring DTs. Knowledge graphs (KGs) have emerged as efficient tools in the industrial domain to efficiently represent data and their relationships from various disciplines in a structured manner. This paper presents an in-situ framework that integrates structured KGs with LLMs for reliable interaction with an in-process DT of a fused deposition modeling 3D printer. The system retrieves both live telemetry and historical data from the DT during operation to generate context-aware responses to user queries during and after printing in the form of an Information Model that contains the printer information. In addition, the 3D simulation environment of the DT is captured using a Virtual Model that contains geometric information including the collision events and motion command history. The proposed framework was shown to generally outperform Retrieval-Augmented Generation and Live configurations for multiple prints, queries, and LLM versions. The framework demonstrates the potential of KG-integrated LLM integration for improving accessibility, interpretability, and decision support in DT-enabled manufacturing environments.

## 1. Introduction

In recent years, smart manufacturing has significantly transformed industrial production through the integration of advanced technologies such as the IoT, cyber-physical systems, and AI [1]. These technologies enable machines to monitor processes, detect anomalies, and support faster decision-making for more flexible, efficient, and adaptive manufacturing systems [2, 3]. Of particular interests in smart manufacturing are digital twins (DTs), which consists of a physical system, its digital model, and bi-directional data exchange between the two [4]. Through process data integration, DTs can evaluate how design and environmental variations affect manufacturing outcomes, while machine learning techniques help handle large sensor datasets and support automated quality control and fault detection during printing [5–7].

However, traditional DT frameworks heavily depend on manual efforts and their operation often demands specialized expertise. These complex interfaces and workflows are significantly challenging to navigate for users to model systems, analyze complex behaviors, and interact effectively with the DT environment. Such complexity therefore limits the accessibility and usability of the DT for engineers, operators, and non-expert users who require rapid understanding and decision-making in dynamic manufacturing environments. These challenges are the main motivation in integrating AI into DTs through large language model (LLM) interactions. Integrating LLMs with DTs allows users to understand system behavior, issue natural language commands, and simplify DT analysis of complex manufacturing systems with reduced technical expertise [8].

Recent advances in LLMs have improved their reasoning to provide new opportunities in data-driven manufacturing with DTs [9]. For instance, Gautam *et al* developed an IIoT-DT framework using LLM agents for real-time monitoring and fault diagnosis [10]. In addition, Chen *et al* proposed a DT–LLM framework for Industry 5.0 focused on architectural integration without experimentally validating real-time capabilities [11]. To assist with data retrieval in DTs, Retrieval Augmented Generation (RAG) aims to address hallucinations in LLMs by incorporating external knowledge into LLM reasoning [12, 13]. However, traditional RAG systems rely on static datasets [14], which are not applicable towards in-process industrial use that require both past and live data for timely decisions. Therefore, streaming RAG has been presented as a possible alternative by enabling continuous retrieval and reasoning. For instance, Kasai *et al* introduced dynamic RAG to update responses with user queries [15]. In addition, Jeon *et al* developed a real-time RAG-LLM for CNC programming, though its implementation was not used with DTs [16]. However, RAG in general treats knowledge as unstructured and independent snippets rather than a connected system, which limits their reliability, technical usability, and accuracy.

Knowledge graphs (KGs) provide an alternative to structured representation in describing real-world domain specific knowledge through entities, attributes, and relationships with ontologies [17–19]. Specifically, ontologies define domain concepts, while KGs integrate heterogenous data to enable sematic querying and reasoning [20]. KGs therefore enable efficient relational querying through graph traversal such as breadth first search, depth first search, structured query language, and embedding based retrieval, for in-process decision making in manufacturing. Bram Steenwinckel *et al* proposed a framework that links sensor-generated events with contextual entities using KG to track activities [21]. Zheng *et al* also demonstrated a KG with a multi-agent reinforcement learning framework to enable self-adaptive decision-making in cognitive manufacturing networks [22]. In addition, multi-layer KG with DTs have been organized using manufacturing data, equipment, process, and their relationships [23]. For instance, Stavropoulou *et al* developed structured relationships with DT data streams for processing monitoring [24]. However, the usage of KGs for in-process LLM requesting for DTs are still lacking. Therefore, there is potential to realize an in-process LLM requesting framework to interface with manufacturing DTs through the use of KGs.

In this paper, we propose an in-process knowledge-based LLM conversational DT framework for manufacturing processes. The framework continuously acquires in-process data from the DT to generate context aware natural responses to user requests. By leveraging structured knowledge representation, the LLM interprets user queries and retrieves relational information to provide meaningful insights into print workflow, system health, performance, collision detection and movement control. This framework supports improved process understanding, intelligent interaction, and reliable monitoring of manufacturing operations.

Therefore, the main contributions of the paper are as follows:

1. Implementation of an ongoing context aware conversational DT framework that integrates information and simulation KGs using live and historical telemetry data.
2. Proposing a simulation-based KG that supports motion command execution, collision detection for safe interactive control within the simulation environment
3. In-process experimental evaluation of the unified framework to assess effectiveness, simulation performance, limitations and latency related to print workflow, motion execution, and system health monitoring.

The remainder of this paper is structured as follows. The DT of interest, proposed framework, and KG schema are first described. The performance of the proposed approach is evaluated in a fused deposition modeling (FDM) 3D printer. Finally, this paper concludes by presenting key observations on the advantages of the proposed approach, along with its limitations and future research directions.

## 2. Materials and methods

### 2.1. DT overview

The proposed system facilitates a process-aware conversational framework that integrates in-process data acquisition, visualization, simulation, structured knowledge modeling, and LLM-based reasoning. In this work, a DT of a FDM Prusa i3 MK3S+ 3D printer was formulated to evaluate the proposed system. As shown in the figure 1, the printer was connected to a Raspberry Pi, which continuously streams process data to firebase through Octoprint. Telemetry data was continuously collected, organized, and stored to reliable storage of both Live and historical information in the DT. A Firebase databased was used to maintain both live and past telemetry to allow the data to be accessed, visualized, and simulated in different platforms.

**Figure 1.**
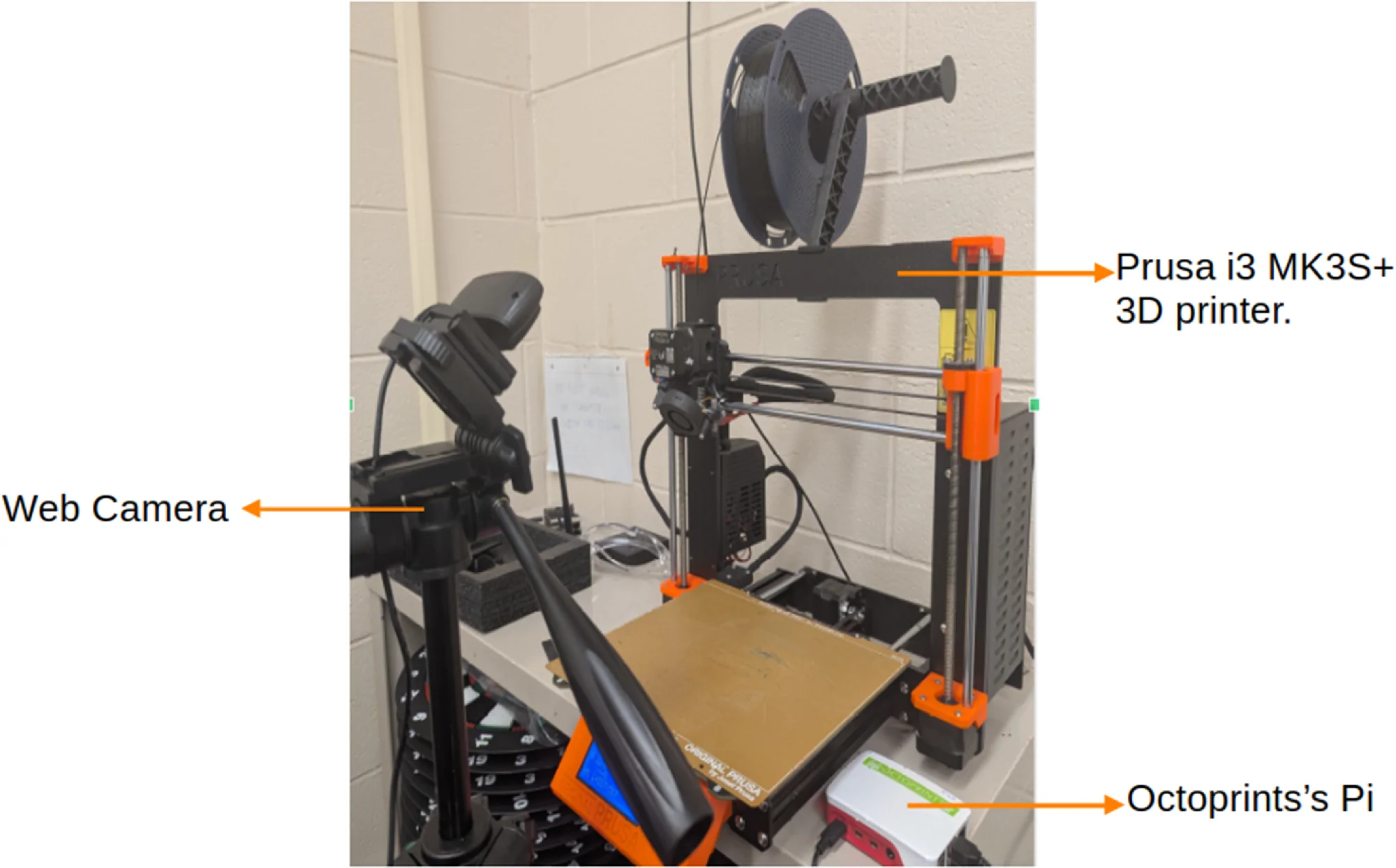
3D printer setup for data acquisition.

A visualization dashboard shown in figure 2 provides an in-process interface for monitoring 3D print progress. The dashboard displays live telemetry data, including print progress, coordinate positions, and printing speed, together with a live print to support continuous process observation. Note that the proposed architecture does not use the visualized dashboard directly, but instead accesses the underlying raw telemetry data. Thus, the dashboard serves primarily as a visual interface for basic process monitoring.

**Figure 2.**
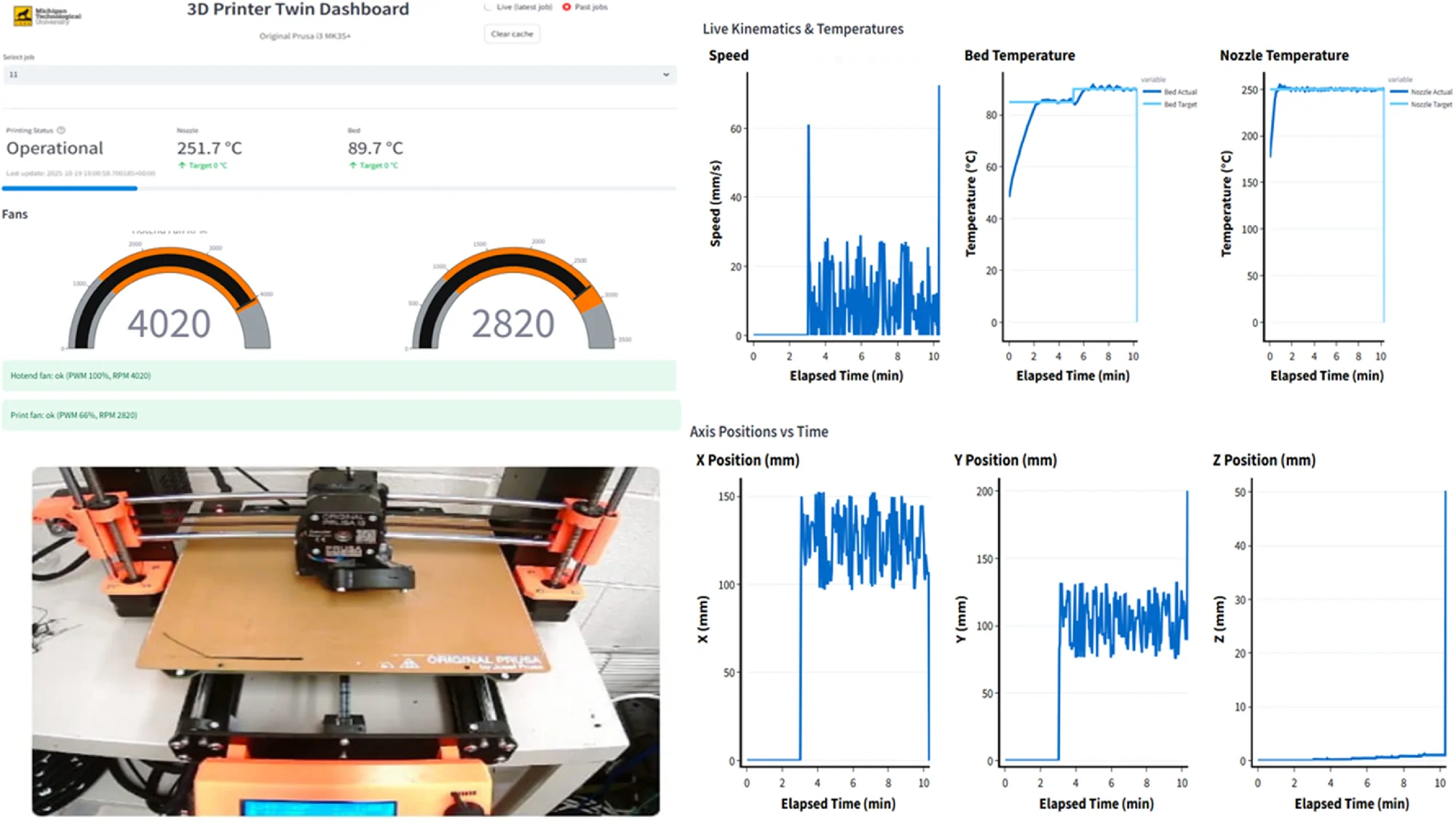
3D printer dashboard.

In addition, a 3D geometry simulation representation of the printer was developed in Unity as a virtual DT as shown in figure 3. The Virtual Model was connected to the printer through the Firebase Unity SDK, which continuously subscribed to the job ID, nozzle coordinates, printing status, print progress, and telemetry timestamp stored in Firebase. The static CAD model was converted into a dynamic component to simulate machine motion during the printing process. The coordinate transformation was implemented to convert telemetry coordinates from *XYZ*–*XZY* to maintain alignment between the physical and virtual system. The received telemetry coordinates were continuously applied to the virtual nozzle to reconstruct print trajectory layer-by-layer to enable spatial motion, path reconstruction, and alignment between the printer and virtual part. Also, to maintain stable visualization for the historical prints, timestamps were bound to a time gap between 20 ms and 250 ms. Additionally, trigger-based physics behavior was integrated into the DT to analyze potential collision detection during printing. Specifically, a trigger collider was attached to the nozzle to detect overlaps with colliders representing the print bed, printed layers, surrounding components and obstacles. Therefore, as the nozzle in Unity moves according to the telemetry data, overlap events are automatically detected and recorded along with the associated spatial coordinates and system components.

**Figure 3.**
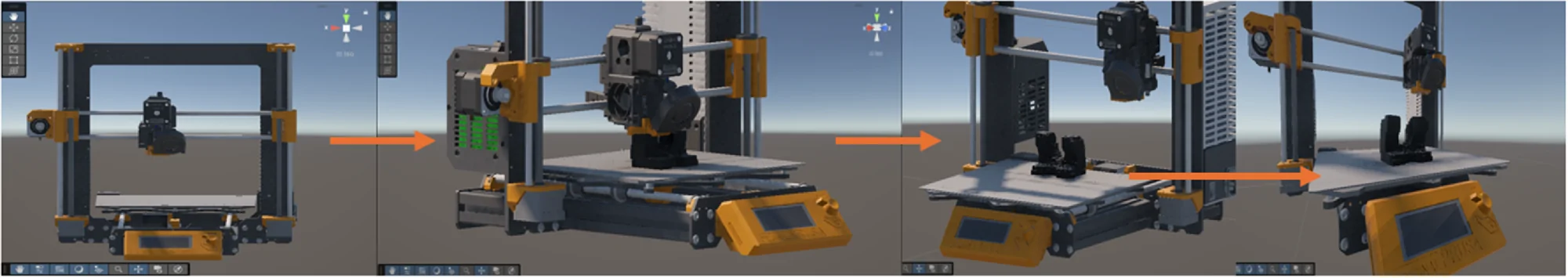
Geometric simulation of the 3D printer.

### 2.2. Decision agent for requests

Figure 4 shows the proposed architecture. In the proposed architecture, a Decision Agent classifies user requests into two categories: information-oriented requests and simulation-oriented requests. KG-1 provides structured printer telemetry (Information Model), while KG-2 supports simulation-based contextual information through collision detection and motion behavior in the Virtual Model. Based on the identified request type, the Decision Agent activates to interact with the required KG to retrieve or compute the required information. Routing is performed using two complementary methods. For descriptive queries related to virtual behavior, the Decision Agent uses a deterministic keyword-based routing strategy via case-insensitive substring matching. Queries containing keywords such as ‘collision events’, ‘virtual environment’, ‘simulation behavior’, ‘motion commands’, ‘unity’, ‘virtual entity’ information are routed to KG-2. Additionally, motion commands, such as ‘Move Y - 50 mm,’ are identified separately using pattern matching based on the axis identifier (X, Y, or Z) and the associated numeric displacement, which are correspondingly routed to KG-2 for execution and logging of collision events. If no keyword matches, the request for any query is directly defaulted to KG-1.

**Figure 4.**
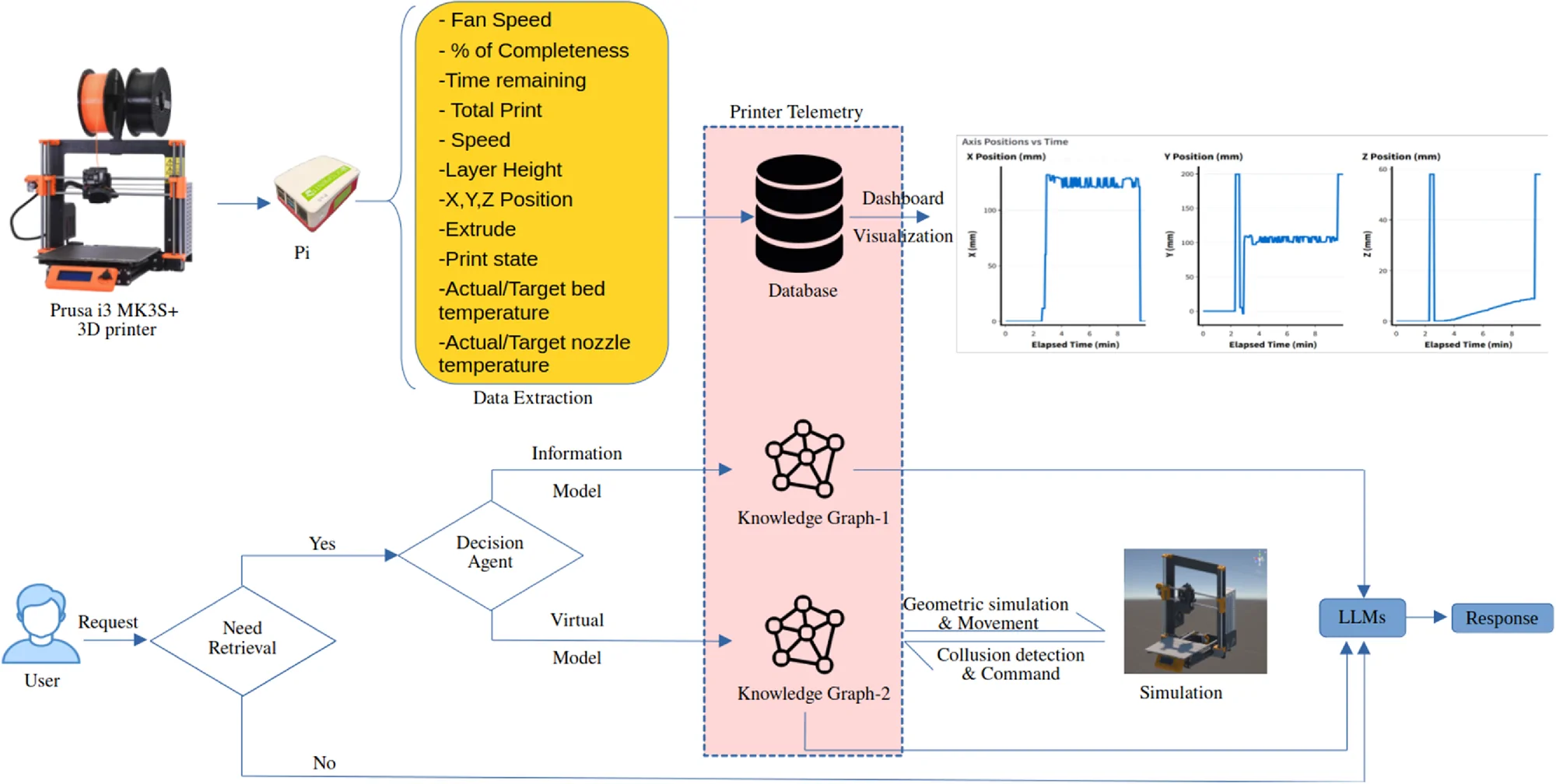
Architecture of the proposed method.

Then the collected information is then sent to the LLM, which generates and returns an appropriate response to the user. The two complementary KGs provide structured contextual information that grounds the LLM’s reasoning and supports the generation of reliable responses. This framework enables user-intent-driven reasoning through a retrieval and decision pipeline that selects between data-level knowledge retrieval and simulation-based reasoning.

### 2.3. Information Model

The Information Model representation is done through ontology that defines the main concepts, their relationships, and associated attributes within the KG. By adding a structured framework into the graph construction, the ontology reduces ambiguity and ensures consistency across data instances, and supports reliable retrieval and analysis of printer behavior.

The resulting KG-1 was implemented and visualized using Neo4j. The core classes of the ontology are as follows:

• **Printer**—Top-level physical entity that anchors all other concepts as the physical AM device executing the printing process.
• **Print job**—Planned and executing process that represents a specific printing tasks from initialization to homing after printing.
• **Live state**—Instantaneous node continuously updated with latest printer values for in-process monitoring as opposed to historical analysis.
• **Telemetry window**—Time-bounded aggregation of measurements at a given timestamp.

Figure 5 shows an instance of the Information Model. Each telemetry time frame is stored as a timestamped telemetry window node connected to its corresponding measurement nodes to preserve temporal relationships across multiple print jobs. Grouping synchronized measurements within a single telemetry window reduces graph complexity while maintaining contextual consistency. Note that the KG-1 maintains dual representations of telemetry data: in-process monitoring and historical analysis. Historical telemetry is stored as time series nodes, while a dedicated live state node is continuously updated with the latest printer values for efficient on-line access. KG-1 is then integrated with a LLM interface to enable natural language querying of both live and historical printer behavior through structured graph retrieval.

**Figure 5.**
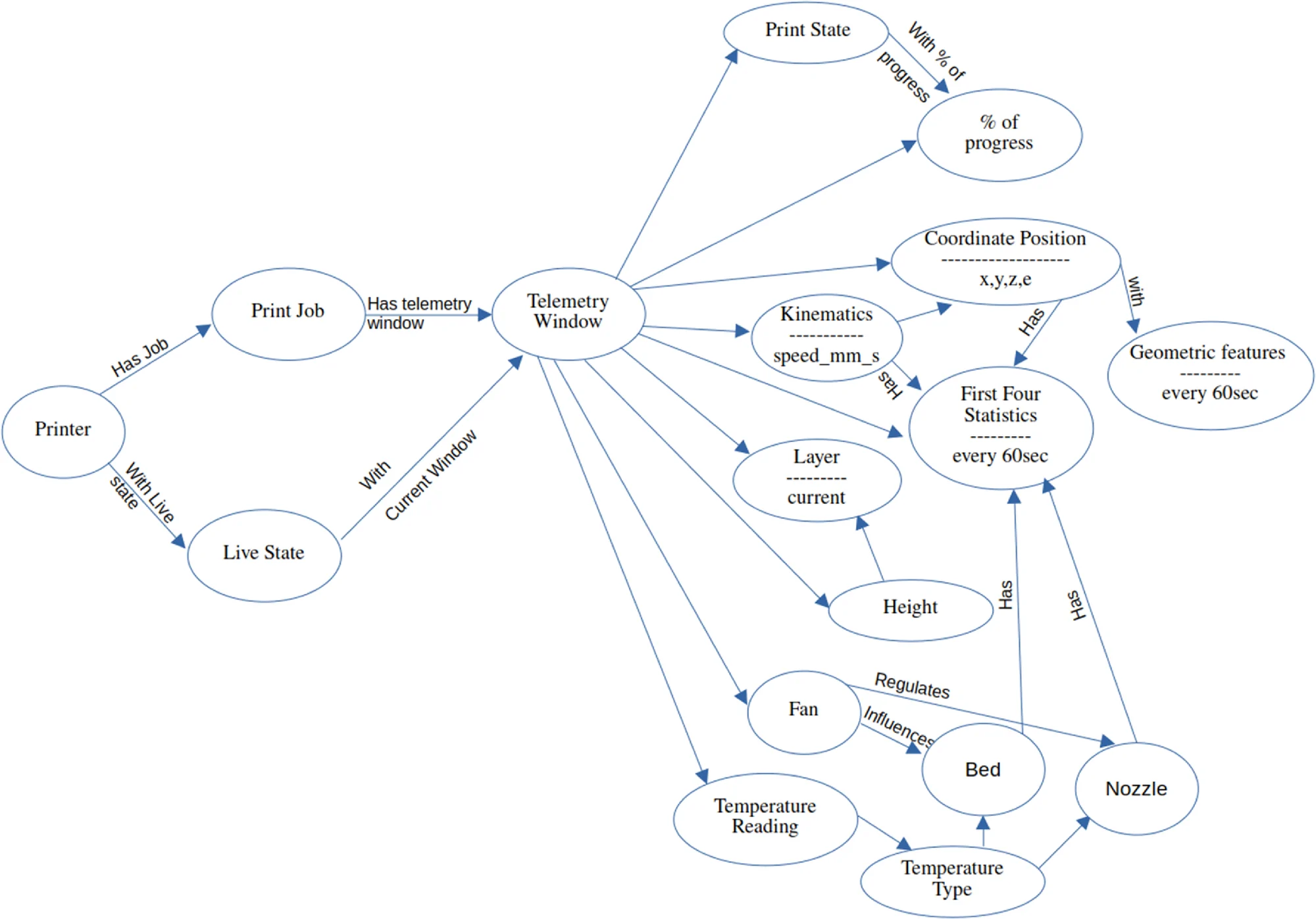
Information Model ontology instance expansion.

Additionally, while storing the telemetry windows, a sliding temporal window of 60 s is used to aggregate process variables such as coordinate position, printing speed, bed and nozzle temperatures. Within each window, the first four statistics (mean, minimum, maximum, and standard deviation) were computed and stored as attributes associated with the corresponding telemetry window node. This aggregation therefore provides insights about the short term printer behavior. Thus, the complete KG-1 enables efficient detection of motion variability, thermal fluctuations, and operational trends across consecutive time windows and for a given instantaneous time.

### 2.4. Virtual Model

In the DT, the virtual environment provides a platform to analyze and experiment with the behavior of 3D physical assets to optimize and enhance in-process productivity within the manufacturing environment. This enables observable motion behavior and interactable dynamics without interpreting the physical process. In this model, a synchronized geometric simulation has been developed in Unity using in-process data. The printer nozzle’s trajectory has been reconstructed in the environment to enable dynamic object spawning that ensures continuous alignment between physical and virtual systems.

This in-process synchronization provides information on printer motion and system behavior that cannot be directly obtained from telemetry alone. For instance, potential interaction issues such as collisions between printer components, printed geometry, and surrounding obstacles can occur without being captured by telemetry. Thus representation of collision scenarios were introduced and analyzed within the Virtual Model. These scenarios include interactions with external obstacles and obstacles with printed objects to enable evaluation of potential failure conditions that may affect printer performance in practical manufacturing environments.

To support this representation, an ontology is developed to define the relationships among printer components, collision contacted objects, positions, instances, and motion commands as shown in figure 6. The core classes of the ontology are as follows:

• **Printer**—Reference node that links collision events to the corresponding physical AM device.
• **Job**—Reference node that connects the simulated motion and collision events to the specific printing task being executed.
• **Collision Events**—Historical node that records collision events detected in the Virtual Model.

**Figure 6.**
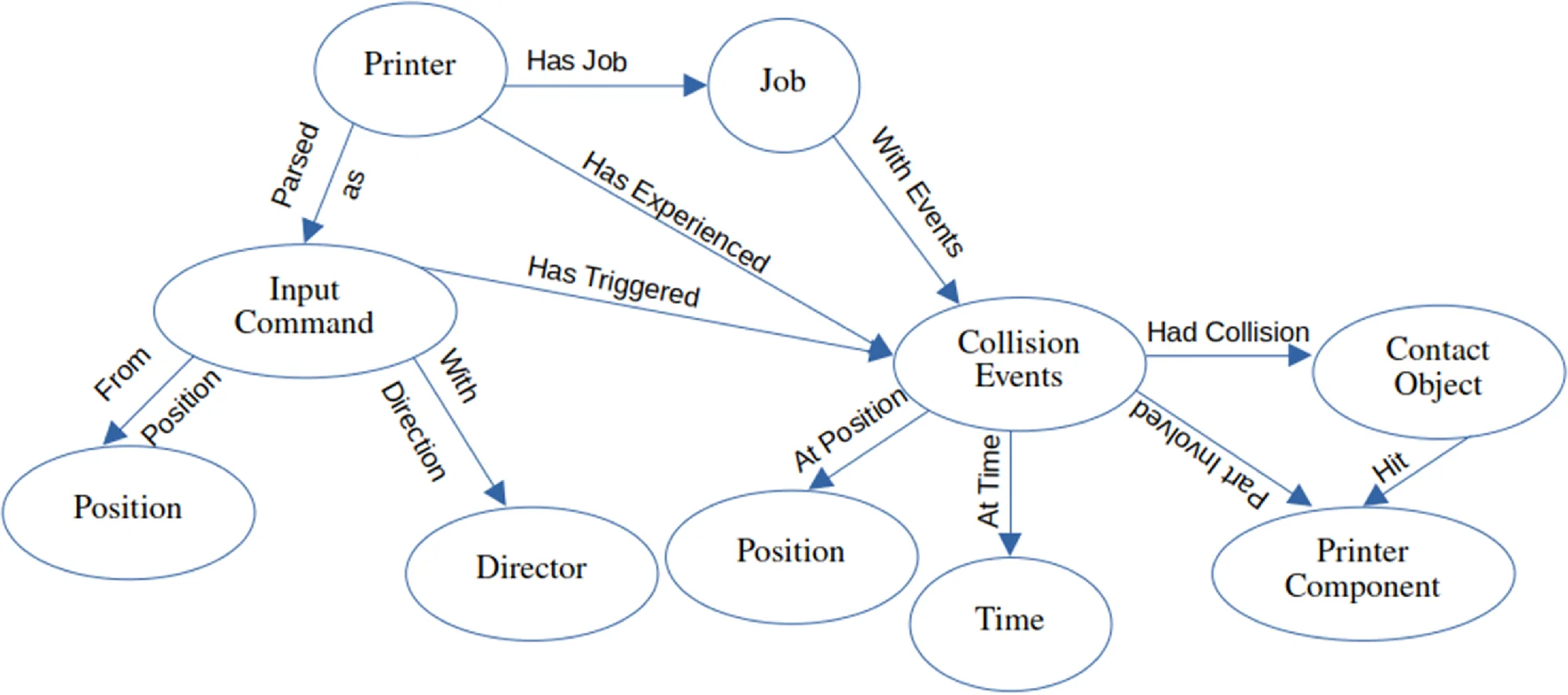
Virtual Model ontology instance expansion.

This work highlights collision detection as the event of interest. Thus the ontology enables LLMs to interact with collision events stored in KG-2. Additionally, LLM-generated motion commands were facilitated with this work to allow interactive analysis of motion behavior within the virtual environment after interaction events occur.

#### 2.4.1. Collision detection

To enable interaction aware reasoning within the virtual environment, a trigger-based collision detection was designed to monitor spatial overlaps between the printer components and the surrounding geometry. Trigger colliders were assigned to the nozzle and extruder assemblies. Unlike the printer bed, the printed layers, the frame structures, and the axis-limit boundaries were represented as tagged interaction surfaces in Unity. Additionally, a cylindrical external obstacle was introduced that deliberately moves within the simulation environment for collision scenarios. Force instance, if an obstacle contacted the nozzle assembly or other printer components, the trigger-based system would detect and overlap the corresponding component pair. Each detected collision event was recorded with its partial position and contact object identity as shown in figure 7. The collision event was stored as a structured instance within KG-2 in the Collision Events node.

**Figure 7.**
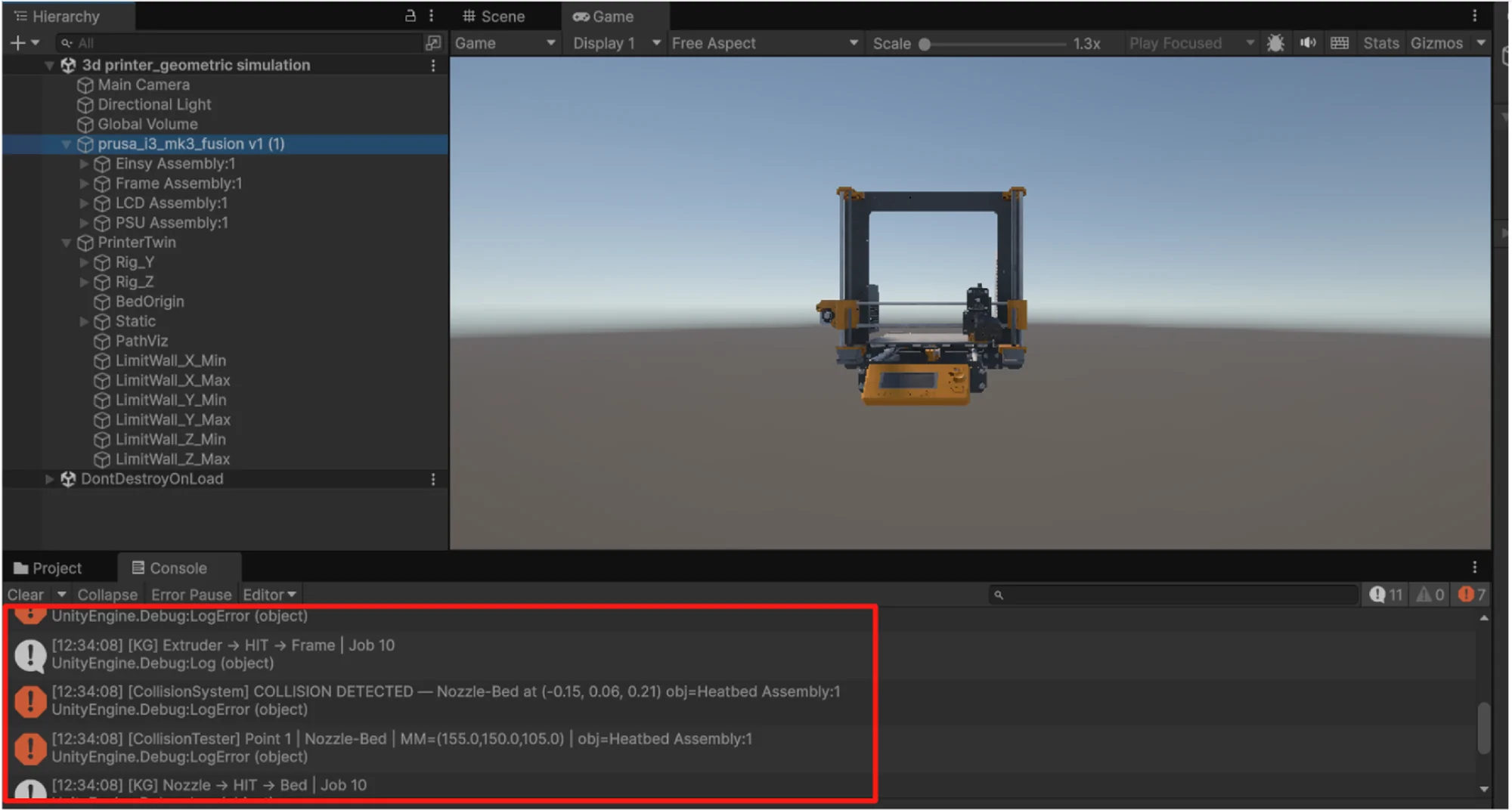
Collision detection being detected by the 3D printer.

#### 2.4.2. Motion commands

Additionally, the framework supports execution of LLM-generated motion commands through the natural language command interface within the constrained limitations of the printer setup. Thus, the displacements are translated into axis-level movements and executed within the simulation environment while collision monitoring remains active. The resulting motion trajectories, collision outcomes, and command execution states were incorporated into the KG-2 to maintain consistency between commanded motion behavior and environmental constraints as shown in figure 8. Thus, the proposed system enables collision-aware reasoning and interactive experimentation within the virtual twin without affecting the physical printer for assisted reasoning for spatial safety assessment and motion feasibility analysis.

**Figure 8.**
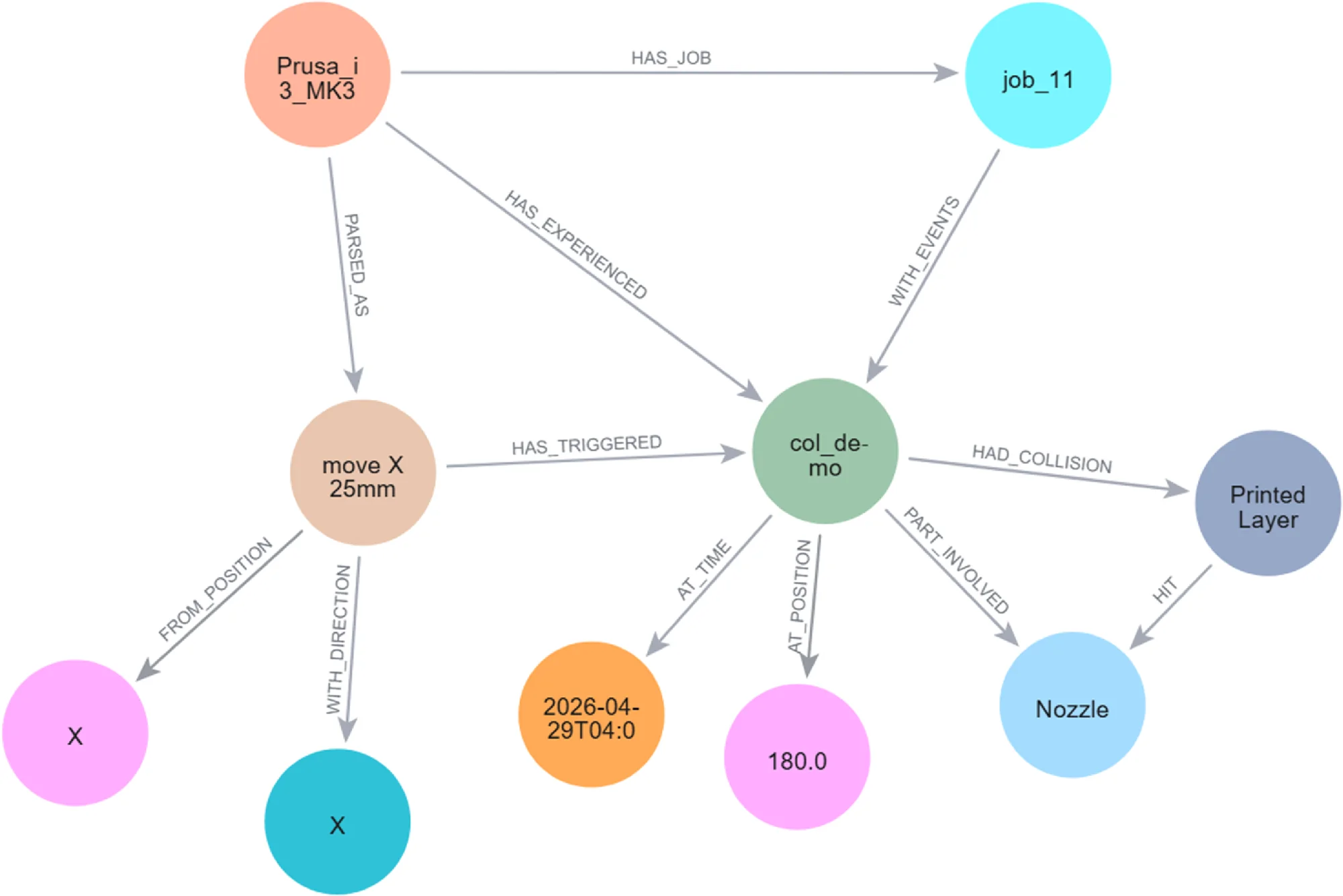
Representative ontology depiction of a collision detection event in Neo4j.

## 3. Experiment setup

### 3.1. Printed test set

To evaluate the performance of the proposed architecture, a total of 50 FDM prints were conducted while the DT was active. Figure 9 shows sample test prints. The print materials included PETG and PLA in this work. Multiple components including frames, brackets, and housings were tested under a variety of print conditions. In addition, the printed test parts were printed with different quality, including acceptable or warped qualities. In this test set, the print duration varied between 10–40 min. Therefore, the test parts of varying shape, material, and quality provided a diverse test set for LLM-based requests of the FDM DT to evaluate the proposed architecture under multiple conditions.

**Figure 9.**
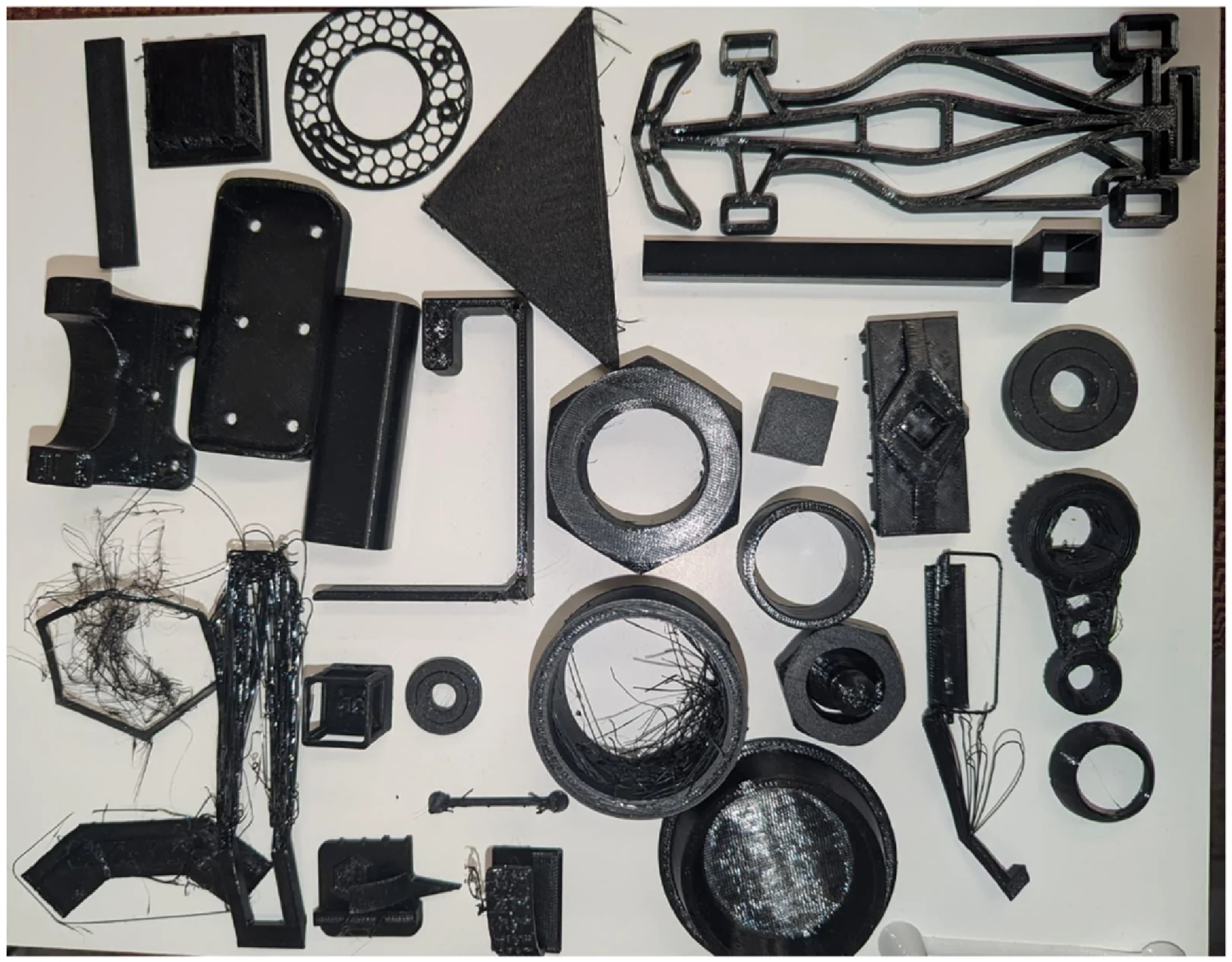
Sample test parts printed for evaluation.

### 3.2. Queries

To evaluate the performance of the LLM to access the Information Model, the queries shown in table 1 were used. The queries are represented as three separate categories. The Current Print queries involve requesting instantaneous data from the DT. The Motion queries involve historical motion of the printer using raw motion telemetry data. The Print Health query refers to the quality of the printer and printed part, which involves inferencing rather than direct data extraction. Therefore, the queries used in this work consider different levels of DT inference capabilities required by the LLM to access the Information Model based on typical questions that end-users can ask. The queries are represented in three categories: Current Print, Motion, and Print Health.

**Table 1.** Queries used for testing the system.

Category	Query	Question text
Current print	Q1	What is the current print status?
	Q2	Provide the current derived acceleration type from the current print.

Motion	Q3	Based on the speed-curve shown in the dashboard image of the current job, is this a slow print or a fast print or undetermined?
	Q4	For the given X, Y, Z graph image of the current job, determine whether the print motion is smooth or jerky or undetermined.

Print health	Q5	Given the printed part image of the current job, is it a successful or failed print or undetermined?

## 4. Results

### 4.1. Information Model metrics

The proposed Information Model performance was evaluated across multiple print queries to assess accuracy with respect to the proposed KG framework, a 60 s sliding window RAG configuration, and live-only data (GPT-Live). In this paper, GPT-5-mini and GPT-4-mini were integrated with these three scenarios and have been performed under similar live conditions. These LLM models were accessed through Open API using Python OpenAI client. Additionally, This testing setup was conducted to analyze, observe, and understand the performance across different configurations to identify the most effective configuration for decision support scenarios in a controlled scenario.

In this work, the Live configuration involves sending the direct first 5 min direct telemetry to the LLM. The RAG configuration involves extracting features from the processed database and sending it to the request-based LLM for reasoning. For the RAG configuration, this work adopts a vector-based retrieval approach to handle continuous and unstructured telemetry of the 3D printer. From this 60 s window the first to fourth statistical moments (mean, variance, skewness, kurtosis) and geometric feature path (displacement, range, and motion distance) were extracted. These summarized windows were converted into vector embeddings using the sentence transformer model all-miniLM-L6-V2. The embeddings were indexed using FAISS through IndexFlatIP index. The embeddings were L2-normalized before indexing which is equivalent to retrieval based on cosine similarity. For each user query, the same embedding model was used to generate the query embedding, and the top five most relevant telemetry windows were retrieved and provided as context to the LLM. These features aim to provide the LLM with both steady state and transient behavior of the ongoing print. These features aim to provide the LLM with both steady state and transient behavior of the ongoing print. For KG configuration the telemetry is structured in Neo4j and retrieved through Cypher queries with integration with LLM. Note that no model fine-tuning was performed and all the models were used through prompt-based API inference.

To evaluate the performance, accuracy, F1 score, precision, and recall were collected for all query types across the three configurations. Additionally, latency was measured as the time delay between sending a prompt and receiving the response. In this paper, accuracy refers to whether the predicted response is correct or wrong when compared to the ground truth. In this work, the ground truth was the collected 60 s information and computed directly offline. For the five queries, the ground truths were defined as follows:

• **Q1**—Most recent printer state from time window.
• **Q2**—Computed type of acceleration from the temporal derivative of the print speed.
• **Q3**—Slow print was defined as being less than 25 mm s$ ^{-1}$.
• **Q4**—Smooth print was defined as being less than 10 mm s$ ^{-2}$.
• **Q5**—Manually labeled print as failed or successful based on visual inspection.

Table 2 shows the metrics for all the LLM configurations. With respect to querying the current state of the print, Q1 and Q2 results are presented as follows. For Q1, the results show that the KG-integrated configuration achieved perfect performance for both GPT-4 and GPT-5 with F1 score, recall, precision, and accuracy all equal to 1.00. This indicates that the KG captured the entity relationships to allow the model to correctly identify the current state of the FDM print. In contrast, the RAG configuration performed poorly for both GPT-4 and GPT-5, with F1 scores of 0.18 and 0.17, respectively. This suggests that text retrieval alone was not sufficient for process monitoring. Interestingly, the GPT-Live configuration for GPT-5 also perfect performance as the KG configuration, though the GPT-4 Live configuration achieved moderate performance with an F1 score of 0.63. This suggests overall reliability depends more heavily on the underlying model and that the KG approach provides robust performance across both GPT versions. For Q2, all configurations produced lower scores than in Q1, which indicates that reporting the current derived acceleration value is a more difficult than classifying print status. The best Q2 performance was achieved by GPT-5 with KG, with an F1 score of 0.56, which shows that the KG improved access to and interpretation of the acceleration feature. The RAG configuration was the weakest overall, which suggests inconsistent retrieval of the correct current acceleration value.

**Table 2.** Metrics for all LLM configurations.

		GPT-4			GPT-5		
Query	Metric	KG	RAG	Live	KG	RAG	Live
Q1	F1 Score	1.00	0.18	0.63	1.00	0.17	1.00
	Recall	1.00	0.20	0.67	1.00	0.19	1.00
	Precision	1.00	0.17	0.59	1.00	0.16	1.00
	Accuracy	1.00	0.50	0.80	1.00	0.48	1.00

Q2	F1 Score	0.46	0.34	0.46	0.56	0.15	0.35
	Recall	0.54	0.47	0.50	0.65	0.18	0.39
	Precision	0.53	0.75	0.52	0.60	0.35	0.41
	Accuracy	0.48	0.30	0.58	0.55	0.25	0.55

Q3	F1 Score	0.33	0.39	0.32	0.33	0.49	0.22
	Recall	0.33	0.33	0.31	0.32	0.49	0.17
	Precision	0.33	0.48	0.33	0.33	0.49	0.33
	Accuracy	1.00	0.65	0.91	0.97	0.95	0.51

Q4	F1 Score	0.60	0.36	0.09	0.65	0.33	0.30
	Recall	0.63	0.41	0.05	0.65	0.26	0.27
	Precision	0.59	0.48	0.33	0.64	0.52	0.33
	Accuracy	0.91	0.51	0.14	0.97	0.37	0.77

Q5	F1 Score	0.50	0.42	0.41	0.54	0.57	0.48
	Recall	0.48	0.39	0.35	0.54	0.56	0.42
	Precision	0.55	0.59	0.62	0.54	0.59	0.57
	Accuracy	0.69	0.54	0.52	0.81	0.85	0.62

To examine the capabilities of the GPT configurations describing historical motion data, Q3 and Q4 were examined. Note that the dashboard speed curve and X, Y, and Z motion plots were visual representations of timestamped telemetry. During evaluation, these images were provided as visual inputs together with the corresponding timestamp-aligned telemetry retrieved through the Live, RAG, or KG configurations. For Q3, the results suggest that speed-based classification from the dashboard image is more challenging than the earlier print-status query because the model must map the visual speed curve to a qualitative label. The strongest F1 performance was 0.49, achieved by GPT-4-RAG, which indicates possible class imbalance or limited class-specific discrimination. The KG configurations showed the best accuracy with 1.00 for GPT-4 and 0.97 for GPT-5. The discrepancy between the high accuracy and low F1 scores in KG demonstrates a class imbalance in the Q3 dataset. This shows that the models were more effective at predicting the dominant speed category. Additionally, even with these imbalances, the high accuracy of the KG configuration indicates that combining the visual plots with timestamp-aligned structured telemetry enabled more reliable motion interpretation. For Q4, the KG configurations provided the strongest and most consistent performance. The KG configuration for GPT-5 achieved the best overall results with an F1 score of 0.65, recall of 0.65 and accuracy of 0.97, while GPT-4-KG followed closely with an F1 score of 0.60 and accuracy of 0.91. This indicates that the KGs are more effective for motion-quality assessment, which is because smoothness or jerkiness can be linked to structured motion features captured by the KG. In contrast, RAG showed weaker performance, which suggests that retrieval did not consistently provide the temporal or geometric context needed. Interestingly, the Live configuration was highly inconsistent with GPT-4 performed poorly with an accuracy of 0.14 while GPT-5 improved accuracy to 0.7. Overall, Q3 and Q4 support the KG approach for graph-based motion interpretation when the task depends on motion trajectories and process behavior rather than just visual inspection.

Although Q3 and Q4 appear similar at the query level, the difference lies in the type of classification being performed. For Q3, the speed-related information available from the data and the dashboard is generally sufficient for determining whether the print can be categorized as slow or fast. In contrast, Q4 requires a more detailed motion analysis to determine whether the movement is smooth or jerky. This involves extracting the X, Y, and Z coordinate trajectories and computing motion-related features such as velocity variations to characterize the quality of the movement. The additional temporal and geometric complexity associated with Q4 result in a more challenging classification task and resulting lower corresponding accuracy and F1 scores.

To examine the ability of the GPT configurations to assess print health based on historical data, Q5 was examined. The results show that the RAG configuration for GPT-5 was the highest performing with an F1 of 0.57, though the KG configuration for GPT-5 was approximately similar with an F1 of 0.54. In addition, the KG configuration for GPT-4 performed the best out of the GPT-4 configurations with an F1 of 0.50. The Live configurations seemed to perform the worst for GPT-5 and GPT-4. Therefore, because of the similarities in performance between KG and RAG configurations for GPT-5 but the better KG performance in GPT-4, this result shows that the KG configuration for GPT-4 had the overall best performance.

Hallucination occurs when generated content appears nonsensical or unfaithful to the provided source content. In the proposed framework, the prompts were tightly constrained using live telemetry, retrieved context, dashboard images. Therefore, broad open-ended hallucinations were not observed during the experimental setup. However, the models occasionally produced definite responses that contradicted the corresponding ground-truth labels. These responses were treated as context inconsistent hallucinations and were quantitatively evaluated using the hallucination rate. The hallucination rate was determined as the ratio of factually incorrect responses to the total number of correct and incorrect responses, which excludes undetermined responses. The hallucination analysis was only evaluated for only Q3, Q4 and Q5 as these questions allowed three possible response categories: correct, incorrect, and undetermined. Here, undetermined means the model did not provide a definitive answer that could be classified as either correct or incorrect. Thus, the undetermined responses were excluded when calculating the conditional incorrect-response rate. Additionally, Q1 and Q2 were not evaluated for hallucinations because incorrect responses for these queries were equivalent to a retrieval or computation error and were already fully captured by the accuracy and F1 scores.

Table 3 shows the hallucination rate over all configurations. Table 3 shows that the KG configuration consistently achieved lowest hallucination rates across all the three queries on both GPT versions. While the RAG and Live configurations showed substantially higher and more variable rates, reaching 0.49 for GPT-5 Live on Q3. These results confirm using structured KG context grounded the LLM responses relatively to reduce the hallucination rate.

**Table 3.** Hallucination rate for LLM configurations for Q3, Q4, and Q5.

	GPT-4			GPT-5		
Query	KG	RAG	Live	KG	RAG	Live
Q3	0.00	0.35	0.11	0.00	0.05	0.49
Q4	0.86	0.42	0.38	0.03	0.22	0.37
Q5	0.12	0.18	0.12	0.14	0.12	0.14

### 4.2. Information Model latency

In addition to the metrics described in tables 2 and 3, the latency performance for each query was studied. The results are show in table 4 along with the pairwise comparison of Games-Howell post hoc test results. For Q1, the latency results show that the fastest response was produced by the KG configuration for GPT-4 with a latency of 0.73 s. The KG configurations were shown to be generally efficient, with the KG configuration for GPT-5 also responding reasonably in 2.02 s, which shows that the structured KGs can support accurate print-status identification without introducing large latency. In contrast, the RAG configuration showed the highest latency, especially for GPT-5, which required 13.87 s for Q1. This suggests that retrieval-based context generation can introduce significant overhead when answering print-status queries. For Q2, the fastest configuration was again the KG configuration for GPT-4, with a latency of 1.24 s. In addition, the KG configuration for GPT-5 required 2.28 s, which is still within a relatively low response range. Interestingly, the Live configuration for GPT-4 showed the highest latency for Q2 with a value of 8.85 s. This result indicates that the live interaction may become slower when the query requires numerical feature reporting rather than categorical status identification.

**Table 4.** Latency (seconds) across LLM configurations *(Mean* ± *Standard Deviation)* with Games-Howell post hoc test results.

GPT-4							
Query		KG		RAG		Live	
Q1	Latency	0.73 ± 0.63		2.87 ± 2.36		2.29 ± 1.88	
	Pairwise	RAG	Live	Live	KG	KG	RAG
	P-value	<0.001	<0.001	0.451	<0.001	<0.001	0.451

Q2	Latency	1.24 ± 1.38		2.05 ± 0.44		8.85 ± 13.63	
	Pairwise	RAG	Live	Live	KG	KG	RAG
	P-value	0.002	0.003	0.009	0.002	0.003	0.009

Q3	Latency	0.78 ± 0.28		2.21 ± 0.31		5.63 ± 5.60	
	Pairwise	RAG	Live	Live	KG	KG	RAG
	P-value	<0.001	<0.001	0.003	<0.001	<0.001	0.003

Q4	Latency	1.06 ± 0.40		0.66 ± 0.14		0.86 ± 0.43	
	Pairwise	RAG	Live	Live	KG	KG	RAG
	P-value	<0.001	0.109	0.036	<0.001	0.109	0.036

Q5	Latency	1.21 ± 0.97		2.09 ± 6.30		1.55 ± 1.09	
	Pairwise	RAG	Live	Live	KG	KG	RAG
	P-value	0.765	0.466	0.904	0.765	0.466	0.904

GPT-5							
Query		KG		RAG		Live	

Q1	Latency	2.02 ± 1.50		13.87 ± 6.87		0.92 ± 0.34	
	Pairwise	RAG	Live	Live	KG	KG	RAG
	P-value	<0.001	<0.001	<0.001	<0.001	<0.001	<0.001

Q2	Latency	2.28 ± 0.87		1.85 ± 0.31		1.35 ± 0.98	
	Pairwise	RAG	Live	Live	KG	KG	RAG
	P-value	0.013	<0.001	0.010	0.013	<0.001	0.010

Q3	Latency	2.59 ± 0.68		2.14 ± 0.67		6.01 ± 5.95	
	Pairwise	RAG	Live	Live	KG	KG	RAG
	P-value	0.018	0.005	0.002	0.018	0.005	0.002

Q4	Latency	5.80 ± 1.83		1.05 ± 0.16		2.01 ± 0.89	
	Pairwise	RAG	Live	Live	KG	KG	RAG
	P-value	<0.001	<0.001	<0.001	<0.001	<0.001	<0.001

Q5	Latency	1.91 ± 1.46		1.22 ± 0.43		3.04 ± 0.98	
	Pairwise	RAG	Live	Live	KG	KG	RAG
	P-value	0.069	0.006	0.000	0.069	0.006	0.000

For Q3, the latency results show that the KG configuration for GPT-4 produced the fastest response with a latency of 0.78 s. The Live configurations were consistently the slowest for this query. This indicates that interpreting the speed curve from the live dashboard image introduced a larger response time than accessing structured KG information or retrieved context. Interestingly, the latency trends significantly differ from Q4 compared to Q3. For Q4, the fastest response was produced by the RAG configuration for GPT-4 with a latency of 0.66 s, followed by the Live configuraiton for GPT-4 at 0.86 s. However, these faster configurations did not necessarily correspond to the best classification performance as shown in table 2. Specifically, the best Q4 performance was achieved by the KG configuration for GPT-5, but it also had the highest latency at 5.80 s, which might suggest that the Q4 KG-integrated motion interpretation required more processing time. Notably, the KG configuration for GPT-4 provides a useful balance with a strong Q4 performance while maintaining reasonable latency.

For Q5, all GPT-4 configurations’ latencies were not statistically significant, as evidenced by the high p-values for all configurations. For GPT-5, the RAG configuration had the best latency, though it was not statistically significant from the KG configuration with a p-value of 0.069. Therefore, from a latency perspective, both the KG and RAG configurations were not statistically significant, though they both overperformed the Live configurations. Therefore, the overall results demonstrate the superior performance of the KG configuration proposed in this work.

### 4.3. Virtual Model command execution

To evaluate KG-2 and its interaction with command executions, motion experiments were conducted where motion commands were sent to the LLM and then reflected in the Unity environment. Motion plots of three example test cases of the motion experiments are shown in figure 10. Figure 10 shows that the LLM interface converts natural language motion commands into printer movements while the KG records the commanded and actual motion history. Movement commands were sent in the syntax form of providing the ‘Move’ keyword followed by the axis and the corresponding distance of motion along the commanded axis. Note that the difference between the step reference and the gradual actual response reflects the physical motion execution time of the printer modeled in Unity.

**Figure 10.**
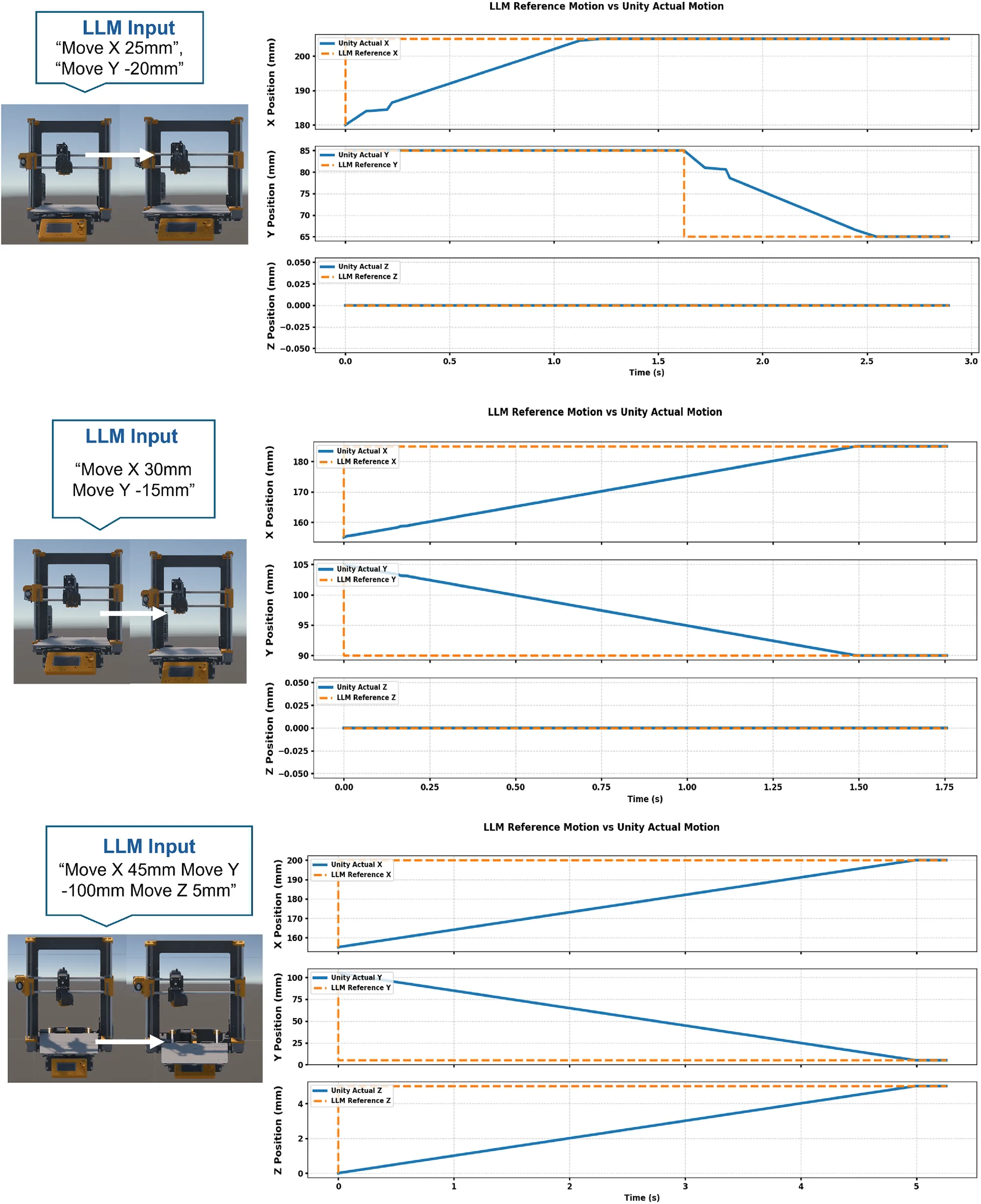
Motion plots for the LLM input movement commands.

To evaluate command execution performance for a variety of test cases, the command parsing success ratio and task completion success ratio were evaluated. Command parsing success refers to the ratio of the natural language commands that were translated correctly into the right structured motion command to all natural language commands given. Task completion success refers to the ratio of success cases when the printer reached its intended target position within the acceptable tolerance out of all commands. To evaluate these metrics, 15 test cases consisting of single-axis, two-axis, and three-axis movement types were conducted, with five different motion commands per movement type. The movement commands intended to test command execution scenarios for all the three X, Y, and Z axes on the printer. Note that commands used in the evaluation were within the predefined positional tolerance and travel limits of the physical printer to ensure that all motions were achievable. The test commands are presented in table 5.

**Table 5.** Motion commands used to evaluate command parsing and task completion.

Test case	Movement type	Movement command
1	Single-Axis	Move X 25 mm
2	Single-Axis	Move Y −20 mm
3	Single-Axis	Move Z 10 mm
4	Single-Axis	Move X −45 mm
5	Single-Axis	Move Z −15 mm

6	Two-Axis	Move X 30 mm, Move Y −15 mm
7	Two-Axis	Move Y 20 mm, Move Z 10 mm
8	Two-Axis	Move X −20 mm, Move Z −10 mm
9	Two-Axis	Move X 15 mm, Move Y 15 mm
10	Two-Axis	Move Y −25 mm, Move Z 5 mm

11	Three-Axis	Move X 45 mm, Move Y −100 mm, Move Z 5 mm
12	Three-Axis	Move X −30 mm, Move Y 20 mm, Move Z −8 mm
13	Three-Axis	Move X 20 mm, Move Y 20 mm, Move Z 20 mm
14	Three-Axis	Move X −15 mm, Move Y −15 mm, Move Z 10 mm
15	Three-Axis	Move X 35 mm, Move Y −10 mm, Move Z −5 mm

Table 6 shows the results of the command execution metrics. Across all 15 test cases, both the GPT-4 and GPT-5 configurations achieved a command parsing success ratio of 1.00 and a task completion success ratio of 1.00. Every natural language command was correctly translated into the intended structured motion command, and the virtual printer successfully reached the specified target position within the predefined tolerance. This result is to be expected since the commands were structured in an orderly manner that always resulted in correct command execution.

**Table 6.** Command execution metrics for KG-2 configurations.

Metric	GPT-4	GPT-5
Command parsing success ratio	1.00	1.00
Task completion success ratio	1.00	1.00

### 4.4. Virtual Model collision detection

To test the capabilities of collision detection by the Virtual Model, this work conducted collision experiments for 11 test cases. The collision events were simulated in the Virtual Model to evaluate the ability of the KG-integrated LLM to record abnormal motion conditions involving physical interference with the printer workspace. As shown in figure 11, three representative obstacle contact scenarios were considered: collision with an externally mounted camera, nozzle collision with a previously printed part, and scraper interference during printer motion. In addition, natural language command executions and print job motions were tested.

**Figure 11.**
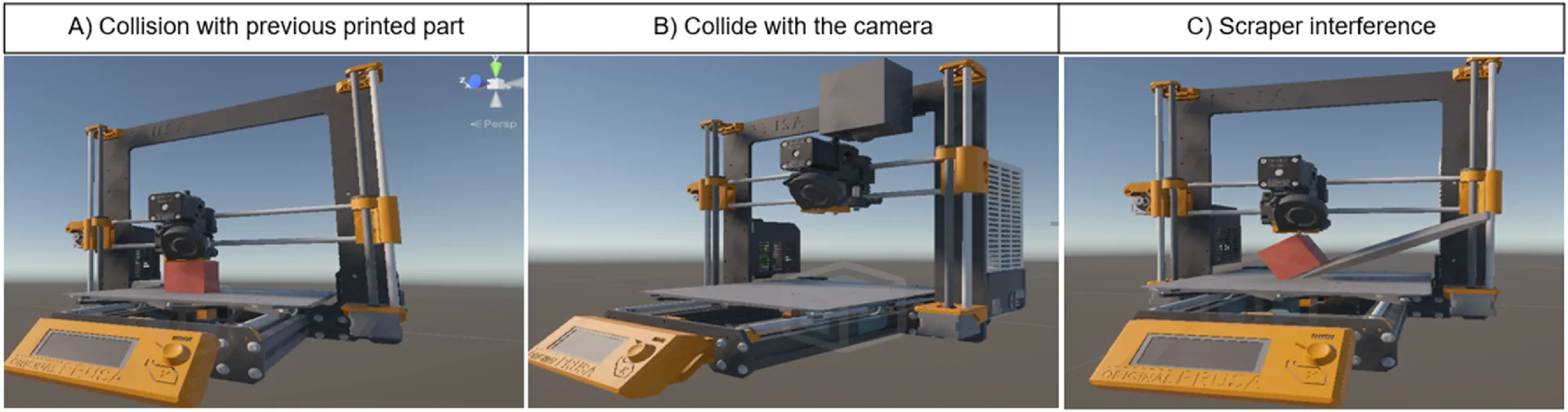
Collision detection contact objects.

In this work, obstacle detection was evaluated as a binary classification. For each interaction between a printer component and a known obstacle, the system must classify the event as ‘collision’ or ‘no collision’. For each test case, the system prediction and the ground-truth label were established independently. The detected outcome was obtained from Unity’s physics-based trigger and colliders pipeline, which includes OnTriggerEnter, NozzleTrigger, TriggerRelay, and PrinterCollisionSystem. When a printer component collider intersected an object tagged as an obstacle, the system recorded a collision event in KG-2. Table 7 shows the test cases used in obstacle detection.

**Table 7.** Scenarios used to evaluate collision detection.

Test case	Contact object	Motion source	Collision event
1	No Obstacle	LLM Command	No Collision
2	No Obstacle	Print Job	No Collision

3	Camera	LLM Command	No Collision
4	Camera	LLM Command	Collision
5	Camera	Print Job	Collision

6	Printed Part	LLM Command	No Collision
7	Printed Part	LLM Command	Collision
8	Printed Part	Print Job	Collision

9	Scraper	LLM Command	No Collision
10	Scraper	LLM Command	Collision
11	Scraper	Print Job	Collision

After the test case was run, the LLM was queried in both GPT-4 and GPT-5 configurations. The LLM was expected to retrieve the Collision Events node in KG-2 and report whether a collision event occurred. Therefore, the result outcomes were compared with the ground-truth labels to calculate accuracy, precision, recall, and F1 score to evaluate the KG-2 retrieval. Table 8 shows the results of the collision detection compared with the ground-truth label. Table 8 shows that the KG-2 configurations for both GPT-4 and GPT-5 were able to successfully report collision and no collision events with no misclassifications. This is because the updating of KG-2 is based on deterministic Unity responses, and the LLM-based retrieval of the collision event information is a pure retrieval of the Unity-based detection event with no additional computation.

**Table 8.** Collision detection metrics for KG-2 configurations.

Metric	GPT-4	GPT-5
F1 Score	1.00	1.00
Recall	1.00	1.00
Precision	1.00	1.00
Accuracy	1.00	1.00

Note that the objective of the KG-2 experiments was not to evaluate the performance, but to provide knowledge on all the possibilities that can be potentially done throughout the system’s operation. Hence, the experiments were designed to verify that the framework could consistently preserve motion histories, collision interactions, and abnormal operational events for downstream LLM-based querying and interpretation. By continuously storing these experimental scenarios and collision interactions within KG-2, the framework accumulates semantic knowledge regarding the conditions and actions that lead to abnormal events over time. Such preserved knowledge can support future DT-enabled manufacturing systems by enabling traceability, interpretable reasoning, and the possibility of avoiding similar collision conditions when integrated with the corresponding physical printer system.

## 5. Conclusion

This works presents a KG-integrated LLM framework for in-process requesting with a manufacturing DT of an FDM 3D printer. The framework integrates two complementary KGs in the form of an Information Model that stores live and historical printer telemetry and a Virtual Model that records motion commands, geometric printer behavior, and collision events in the Unity simulation environment. A Decision Agent routes user requests to the appropriate KG and supplies the retrieved context to the LLM. The experimental results show that the KG-integrated configuration provided the most reliable overall performance compared to RAG and Live configurations. The latency and hallucination results further showed that the KG framework consistently maintained practical responses and time periods compared to the RAG and Live configurations. The Virtual Model results also demonstrated that natural language motion commands could be translated into executable axis-level movements along with detection of collision events being stored in the KG for later interpretation. Therefore, the results demonstrate that structured KG framework improves the reliability, interpretability, and traceability of LLM interaction with DTs for FDM process monitoring compared with unstructured and RAG retrieval methods.

Future work includes expanding fault coverage and strengthening closed-loop interaction. Although the KG improved access to answering the queries, the underlying models suggests that future implementations should integrate deterministic querying and calculation tools to ensure exact reporting. In addition, future work includes evaluating the framework across a larger set of process conditions and failure modes, which include more complex queries that require simultaneous multimodal reasoning from images, telemetry, and simulation data. The Virtual Model can also be extended with more realistic collision physics and automated recovery recommendations to allow evaluation of the generated motion commands before being transferred to the physical printer. Furthermore, future studies involve investigating uncertainty estimation in the KG retrieval to support more robust in-process monitoring and safer LLM-DT interaction and implementing different prompt strategies. The methods employed in this work are general and can be applied to other DT applications in manufacturing, such as assembly inspection, or other sectors such as automated driving and health monitoring.

## Data Availability

The data that support the findings of this study are openly available at the following URL/DOI: https://github.com/rkaruka/In-Process-Large-Language-Model-Requesting-through-Knowledge-Graphs-for-Interfacing-.git [25].
